# Feasibility of an Evidence-Based Parent-Mediated Intervention for Autism Spectrum Disorder in a Community Healthcare Service in Italy

**DOI:** 10.3390/children12121651

**Published:** 2025-12-05

**Authors:** Natasha Chericoni, Ilaria Colombino, Eugenia Conti, Giulia Guainai, Benedetta Riva, Lu Qu, Fabio Apicella, Sara Calderoni, Raffaella Tancredi, Andrea Guzzetta, Costanza Colombi

**Affiliations:** 1 IRCCS Stella Maris Foundation, 56128 Pisa, Italy; 2 Department of Clinical and Experimental Medicine, University of Pisa, 56126 Pisa, Italy; 3 Child Health Care Medical Division, Shanghai Children’s Hospital, Shanghai Jiao Tong University, Shanghai 200062, China

**Keywords:** parent-mediated intervention, parent-ESDM, early start Denver model, autism spectrum disorder

## Abstract

**Background/Objectives**: Parental involvement is currently recommended by Italian national guidelines on autism spectrum disorder (ASD) intervention. However, research on the impact of parent-mediated interventions on parental skills and children’s outcomes in Italy is limited. This study evaluated the feasibility of delivering Parent-ESDM (Parent-mediated Early Start Denver Model), a well-supported Naturalistic Developmental Behavioral Intervention (NDBI) known to benefit parents’ well-being and children’s development, within an Italian healthcare service. **Methods**: Twenty parent–child dyads participated in weekly 1 h Parent-ESDM sessions for 6 months. Spontaneous parent–child interactions were assessed at baseline, mid-intervention, and post-intervention to examine parents’ use of NDBI strategies and changes in children’s core ASD behaviors. **Results**: Throughout the intervention, parents acquired a good level of fidelity in the use of NDBI strategies and children obtained significant improvements in core ASD behaviors. **Conclusions**: These preliminary findings support the feasibility of delivering a parent-mediated intervention within an Italian healthcare service. The positive trends observed provide a strong rationale for conducting controlled trials to more definitively evaluate this model and its potential adoption as a future standard practice.

## 1. Introduction

Autism Spectrum Disorder (ASD) is a neurodevelopmental condition characterized by deficits in social communication and restricted and repetitive behaviors and interests [1]. It manifests in early childhood and persists into adulthood, creating multiple challenges for children, families, educational and health services, and generating substantial societal costs [2,3,4]. Research shows that earlier intervention leads to better outcomes [5,6]. However, although signs of ASD can be detected within the first months of life [7,8] and reliable diagnosis is possible by around 14 months [9], diagnosis in clinical practice typically occurs around 4 years of age, with intervention starting even later [10]. Due to the high-intensity treatment recommended and the limited availability of specialized professionals, families often experience long waiting times for public health services [11]. As a result, in many countries, children receive low-intensity, unspecialized treatment at ages when neural plasticity is no longer at its peak [12].

Despite strong evidence supporting the importance of involving parents in intervention to optimize developmental outcomes [13], parent-mediated programs remain scarce in many countries, including Italy [14]. Parent-mediated interventions have the potential to reduce waiting times and increase access to daylong intervention as parents can embed learning opportunities within everyday routines, promoting generalization across environments [15,16,17]. These programs aim to enhance parent–child engagement and support behavior regulation, social communication, and interaction, by increasing parents’ skills and knowledge through a range of methods, such as direct coaching, video-feedback, and didactic approaches in individual or group formats [18]. Parents are taught specific strategies not because of any presumed deficit in parenting, but because autistic children often display subtle communicative acts and atypical behaviors that are difficult to interpret without guidance. As parents are the individuals to whom children respond the most and with whom they spend most time in early development [13,19], supporting parents’ understanding of their child’s divergent development can have broad cascading effects [20,21]. Compared with usual treatment or waitlist conditions, parent-mediated interventions yield moderately strong benefits in children’s social behaviors, language/communication, and maladaptive behavior [18], and also improve parents’ sense of competence [22] and mental well-being, reducing stress and strain [23,24]. Currently, both international and national guidelines recommend including parents in intervention programs [25,26,27].

In Italy, the provision of diagnostic and intervention services varies greatly across regions [28,29]. In most areas, public health services offer autistic children low-intensity, non-specialized interventions such as speech therapy, educational services, and neuropsychomotor therapy [30]. Although national guidelines recommend involving parents in ASD intervention [26], this practice is still not routinely implemented in community services, where therapy is typically delivered without parental presence [14]. To date, only three randomized controlled studies evaluating parent involvement have been conducted in Italy, yielding mixed findings [31,32,33].

The first study, conducted by Valeri et al. [31] involved 34 autistic children aged 2 to 6.11 years who were randomly assigned to either a Low-intensity Psychosocial Intervention (LPI) inspired by TEACCH intervention or LPI combined with Cooperative Parent-Mediated Therapy (CPMT). CPMT aimed to teach parents to support seven target skills in their children: 1. socio-emotional engagement, 2. emotional regulation, 3. imitation, 4. communication, 5. joint attention, 6. play and cognitive flexibility, and 7. cooperative interaction. The CPMT was delivered in a hospital setting over 6 months through 15 sessions of 60 min each, while the LPI was delivered in home and school settings for 4 h per week. Although based on a small sample, findings indicated that children receiving CPMT alongside LPI tended to show greater gains than those receiving LPI alone, with improvements observed in social–communication skills, ASD symptom severity, emotional regulation and parental stress.

A second study by Carta et al. [32] randomized 40 autistic children aged 2–10 years to either Individual Treatment As Usual (TAU) or TAU plus CPMT. Parents in the CPMT group showed indications of more substantial improvements in the quality of parent–child interaction relative to TAU alone. Additional positive trends were also reported for autism symptom severity, adaptive functioning, and parental stress. While exploratory, these findings offer initial support for the feasibility and potential utility of CPMT within community-based services.

A third study, conducted by Salomone et al. [33] randomized 86 children aged 2–5 years to receive either WHO Caregiver Skills Training (CST) or enhanced treatment-as-usual (eTAU, i.e., one psychoeducation session in addition to TAU). The CST program lasted 3 months and included three home visits focused on goal setting, coaching and support for independent practice, as well as nine group sessions covering the following topics: 1. getting and keeping children engaged; 2. building home and play routines; 3. understanding and promoting communication; 4. preventing and reducing challenging behavior; 5. promoting daily living skills; and 6. caregiver well-being and problem solving. The CST was found to be both acceptable and feasible. Parents significantly improved in their ability to scaffold their child’s actions, follow the child’s focus, and appropriately modulate affective communication to enhance interaction. However, no significant differences emerged between groups in children’s joint engagement, availability to interact, and autism symptom severity. Thus, improvements in parents’ interaction skills did not translate into measurable child-level changes within the study timeframe. One possibility highlighted in the literature is that more targeted training, emphasizing individualized coaching with in vivo feedback rather than broader coaching approaches, may be necessary to enhance the impact of parent-mediated interventions on children’s outcomes [34].

Regarding Naturalistic Developmental Behavioral Interventions (NDBIs), considered among the most effective early interventions [35], the Early Start Denver Model (ESDM) is the most studied NDBI within the Italian public health system, although research has focused primarily on therapist-delivered, one-to-one formats. ESDM targets verbal and nonverbal communication, joint attention, social engagement, imitation, play and cognition [36] and has demonstrated significant effects on autism symptoms, language, and cognition in randomized controlled trials [37]. Five Italian community-based studies have evaluated ESDM delivered by therapists [29,30,38,39,40], consistently showing stronger developmental gains in social communication for children receiving ESDM compared with non-specialized treatments. Notably, Devescovi et al. [41] reported that children who received a year of low-intensity individual ESDM maintained their socio-communicative gains during a six-month interruption of in-person services caused by COVID-19, despite receiving only remote parental support. During this period, participants also showed a reduction in restrictive and repetitive behaviors. As telehealth support to parents was the only intervention available, these findings underscore the importance of actively involving parents in their child’s therapy.

Although national guidelines recommend parent-mediated intervention [26], research on its effects on parental skills [31,32,33] and child outcomes [31,32] in Italy remains limited. The overall aim of the present study is to evaluate the feasibility of delivering Parent-ESDM (P-ESDM), a well-supported parent-mediated intervention shown to benefit parental well-being, parenting stress and child development [16,24,42,43,44], within Italian community services. To our knowledge, this is the first study to examine the feasibility of a low-intensity parent-mediated intervention based on the Early Start Denver Model in Italy. We present preliminary results on parents’ use of NDBI strategies and on children’s changes in ASD core symptoms and developmental milestones.

## 2. Materials and Methods

### 2.1. Participants

Twenty children with a confirmed or suspected diagnosis of ASD, due to their young age, participated in this study (M/F = 15/5; age in months: M = 28.25, SD = 7.85, range 14–48). All children had received their clinical diagnosis within community mental health services and were referred to the project to receive an intervention while waiting to begin treatment within community services. The diagnosis was provided by a multidisciplinary team of ASD specialists, including child neuropsychiatrists and psychologists working in community mental health services. Before the start of the intervention, as part of the research project, all children completed a baseline assessment that included standardized developmental measures (Griffiths Developmental Scales III [45]), diagnostic instruments (Autism Diagnostic Observation Schedule-2 [46,47,48]), and adaptive behavior measures (Vineland Adaptive Behavior Scales, Second Edition [49]) to confirm the diagnosis and further characterize their profile. An overview of children’s demographic and baseline characteristics is presented in Table 1.

Children were eligible to participate if they met the following inclusion criteria: (i) 12–48 months of age at the time of enrollment; (ii) a diagnosis of Autism Spectrum Disorder determined by a qualified professional; (iii) agreement to participate in the intervention once a week over a period of 6 months; and (iv) Italian as one of the languages spoken at home (as the research staff were not trained to provide the intervention in other languages). Children were excluded if they presented significant sensory or motor impairments that could interfere with participation in the intervention or with the assessment procedures.

Parents who participated in the study were mainly mothers (18/20). The mean age of mothers was 36.35 years (SD = 5.80), while fathers had a mean age of 39.65 years (SD = 6.44). Most parents had completed at least a high school education (see Table 2 for an overview of parents’ demographic information).

### 2.2. Intervention

Parent–child dyads received the Parent-ESDM [16], an evidence-based specialized intervention for young autistic children in which therapists support parents in implementing ESDM principles. The ESDM uses a child-centered responsive interaction style and 10 foundational intervention themes, including (1) social attention and motivation for learning, (2) sensory social routines, (3) dyadic engagement, (4) non-verbal communication, (5) imitation, (6) antecedent-behavior-consequences relationships, (7) joint attention, (8) functional play, (9) symbolic play, and (10) speech development [36]. Each child’s plan is defined by a set of short-term objectives to be achieved over a 12-week period. The objectives are derived from an assessment based on the ESDM Curriculum Checklist (see the section on Secondary Outcome Measures for a detailed description of the tool), performed by a certified ESDM therapist. The therapist discusses the objectives and intervention strategies with the parent and supports the parent during the therapy sessions. After the first 12 weeks of intervention, a second assessment is conducted using the ESDM Curriculum Checklist to determine new objectives or adjust previous objectives that may not have been reached.

### 2.3. Primary Outcome Measure

#### The Measure of NDBI Strategy Implementation-Caregiver Change (MONSI-CC)

The MONSI-CC [50] is an observational measure of caregivers’ implementation of NDBI strategies. The MONSI-CC coding scheme was applied to 10 min videos of play interaction between the child and the caregiver who participated in the intervention. The video recordings were taken at the clinic, using a standardized set of toys. The MONSI-CC is composed of 20 items that focus on specific strategies, and yields five subdomains (Environmental Set-Up, Child-Guided Interactions, Active Teaching and Learning, Opportunities for Social Communication, and Natural Reinforcement and Scaffolding), and an overall total score. For the coding of the video, the recording is watched twice. The first time, the entire 10 min clip is watched to determine the child’s overall language and play level, and then the video is split into two segments (e.g., 5 and 5 min), which are coded separately and subsequently added to calculate the average level of NDBI strategy implementation across the entire video. Each item is rated on a 1–5 scale (1 is the lowest possible score and 5 is the highest possible score) that is dependent on a combination of (1) the frequency/consistency of the strategy used, (2) the effectiveness, and (3) the number of missed opportunities. Higher scores indicate higher frequency, effectiveness, and appropriateness of NDBI strategies. Coders were required to meet three reliability criteria on three consecutive segments: (i) ≥80% item-level agreement (no more than four items differing by >1 point) within each segment; (ii) ≥80% agreement on each domain score, within each segment; and (iii) ≥90% agreement on total scores.

### 2.4. Secondary Outcome Measures

#### 2.4.1. The ESDM Curriculum Checklist

The ESDM Curriculum Checklist [36] provides a very detailed list of items for ASD-specific social and preverbal communication development arranged hierarchically across a period of 8–48 months. It is administered to children at the start of the intervention, and it is used to construct individualized treatment objectives. The Curriculum Checklist covers the following 8 domains: communication, social interaction, imitation skills, cognitive skills, play skills, fine motor skills, gross motor skills, and independence/behavior. A curriculum assessment is delivered by the therapist prior to the initiation of the intervention and subsequently at 12-week intervals, to develop the treatment objectives to be implemented during the following 12 weeks. During the assessment, the therapist evaluates all the items using direct interaction with the child and parent-reported information. Based on the assessment, two to three objectives are written for each developmental domain. These treatment objectives define the child’s treatment for the next 12 weeks. They are written in an A-B-C format specifying the antecedent condition in which the target behavior will occur, the operational definition of the behavior involved in the targeted skill, and the criteria to determine mastery. Children generally achieve 75–90% of their objectives each quarter. If an objective is not achieved during the quarter, its appropriateness is reconsidered.

#### 2.4.2. Behavior Observation of Social Communication Change (BOSCC)

The BOSCC [51] is a treatment response measure developed to assess changes in core ASD symptoms in young children with ASD. The BOSCC was developed by modifying and expanding codes from the ADOS-2 [46,47], in order to capture more nuanced variation. Items are coded on a 6-point scale from 0 to 5, with higher scores reflecting more atypical behavior. It consists of 12 items that provide a total score, as well as scores in Social Communication (SC) and Restricted and Repetitive Behaviors (RRBs). Inter-rater reliability, short-term test–retest reliability, and sensitivity of change over time using the BOSCC codes have been documented [51]. The BOSCC is coded from 15 min videos of caregiver–child interaction using the same toys (e.g., cause-and-effect toys, musical instruments, construction toys, and pretend-play toys) across dyads and times. Of the 15 min videos, the 10 central minutes, obtained by eliminating the first and last 2.5 min, are coded. The scores of the two 5 min segments are summed and averaged to obtain the total score, as well as the SC and RRB scores. All interactions between the child and caregiver were recorded with the same caregiver (mother or father), who participated in the program. Coding reliability was established when coders met both of the following criteria on six consecutive 5 min calibration segments: (1) item-level agreement within 1 point on >80% of items, and (2) total score agreement within 3 points.

### 2.5. Procedures

The study was carried out at the IRCCS Fondazione Stella Maris (FSM), Pisa, Italy, in accordance with the standards for good ethical practice of the Declaration of Helsinki, and was approved by the Pediatric Ethics Committee of the Meyer Hospital on 9 December 2021. Families enrolled in the project were all on a waiting list for community services in the Tuscany area. Written informed consent was obtained from all the children’s parents. Families enrolled in the project participated in a 6-month parent-mediated intervention based on ESDM principles. During the therapy sessions, the parents were coached by trained therapists to implement the intervention objectives. All the therapists were certified to deliver ESDM therapy and received weekly supervision from an ESDM trainer (CC). The program was delivered at the FSM and involved participating in 1 h sessions, once a week, over a period of 6 months. Data on parents’ use of NDBI strategies and child behavior were collected 1 week prior to the beginning of the intervention (T0), after 3 months from the beginning of the intervention (T1), and at the end of the intervention, in the two weeks following the last treatment session (T2). Assessments were performed by research collaborators who were not involved in the intervention. Parent–child interaction videos were coded by two master’s-level research assistants who had completed training and achieved reliability for MONSI-CC and BOSCC.

### 2.6. Data Analysis

The distribution of the study variables met the assumptions of normality; thus, parametric analyses were conducted. A repeated measures analysis of variance (ANOVA) was used to assess changes in the MONSI-CC, ESDM Curriculum Checklist, and BOSCC at three time points: baseline (T0), 3 months (T1), and 6 months post-intervention (T2). Statistical significance was set at *p* < 0.05.

## 3. Results

### 3.1. Primary Outcome: Changes in Caregiver Implementation of NDBI Strategies

**Figure 1:**
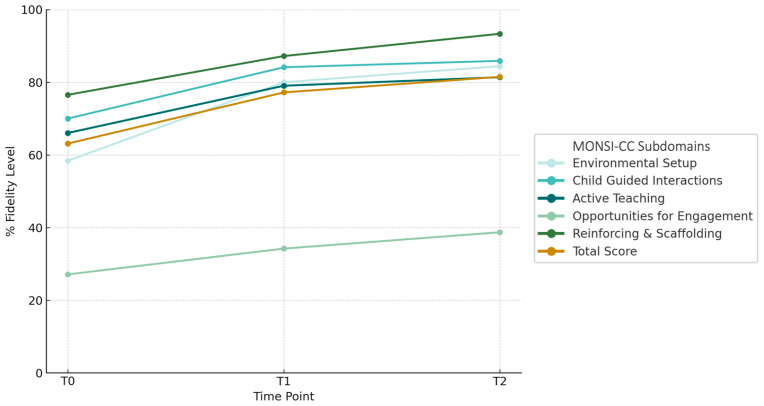
Changes in MONSI-CC subdomains according to the level of fidelity reached in each NDBI strategy. Percentages were calculated relative to the maximum achievable score for each MONSI-CC subdomain.

A repeated measures ANOVA showed a significant increase in the MONSI-CC total score over time, with post hoc tests indicating significant gains from T0 to T1 that were maintained at T2, and no further change between T1 and T2. All MONSI-CC subdomains showed significant time effects, with improvements emerging from T0 to T1 and remaining stable at T2. Detailed statistics for each subdomain and contrast are reported in Table 3. Figure 1 illustrates the trajectory of change across MONSI-CC subdomains.

### 3.2. Secondary Outcomes 

#### 3.2.1. Changes in Children’s Acquisition of Therapy Objectives

**Figure 2:**
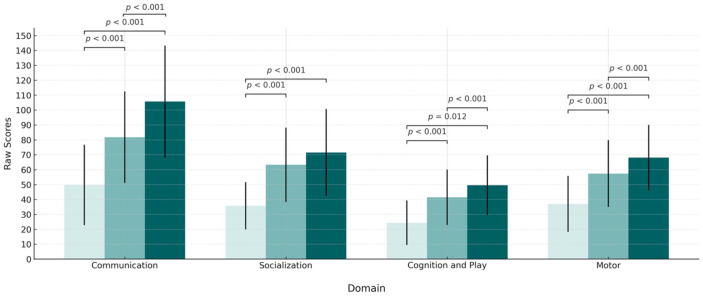
Changes in the ESDM domains over time.

A repeated measures ANOVA showed significant improvements on the ESDM Curriculum Checklist total score over time. Post hoc tests indicated marked gains from T0 to T1 that further increased at T2 across all domains, with the exception of the Socialization domain, which showed no significant change between T1 and T2. Detailed statistics for each domain and pairwise comparison are reported in Table 4. Figure 2 provides an overview of developmental progress across ESDM domains.

#### 3.2.2. Changes in Children’s ASD Core Symptoms

**Figure 3:**
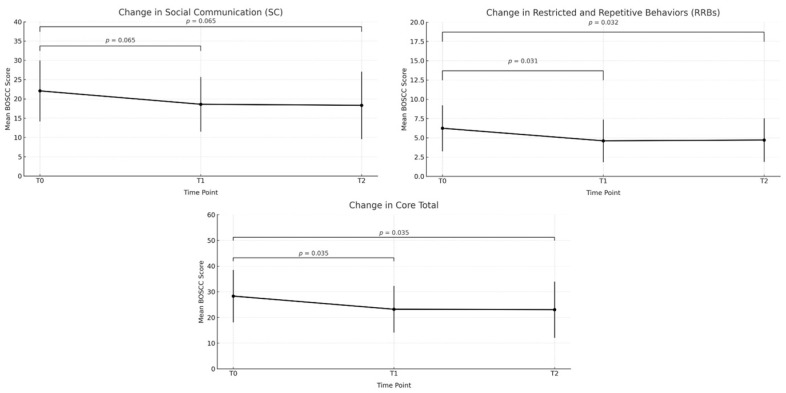
Changes in BOSCC domains over time.

A repeated measures ANOVA showed a reduction in ASD core symptoms on the BOSCC total score, with post hoc tests indicating improvements from T0 to T1 that were maintained at T2, and no further changes were observed between T1 and T2. A similar pattern emerged for both the Social Communication and the Restricted and Repetitive Behaviors domains, with reductions occurring between T0 and T1/T2 and stability thereafter. Detailed statistics for all time point comparisons are reported in Table 5. Figure 3 illustrates changes in BOSCC scores over time.

## 4. Discussion

In this article we report data on the feasibility of a parent-mediated intervention based on ESDM principles, implemented in an Italian community service, in the Pisa area. Children recruited in the study had recently received an ASD diagnosis or were considered at risk for the condition, and were on a waiting list for community services. In line with Italian healthcare procedures, the intervention was delivered in a hospital setting. However, in comparison to the most common therapist–child delivery in Italy, the ESDM intervention was administered within the parent-mediated modality.

In order to evaluate to what extent parents would be able to acquire strategies for stimulating their child’s social communication development, we coded videos of parent–child interaction at the beginning of intervention, after 3 months from the start of the intervention and at the end of the intervention, using the MONSI-CC scale, which measures changes in parents’ implementation of NDBI strategies. In accordance with our expectations, parents mastered foundational skills for supporting their child’s social communication development. Indeed, after only 3 months (T1), we found a significant improvement in Environmental Set-up, Child-Guided Interactions, Active Teaching and Learning, and Naturally Reinforcing and Scaffolding, and after 6 months (T2) we detected an additional significant increase in Opportunities for Engagement. Parents reached at least 80% of fidelity in all the strategies examined (except for Opportunities for Engagement) within the first 3 months of intervention (T1 assessment) and then maintained this positive result at the T2 assessment. The area that remained with a low fidelity level even at T2, namely, Opportunities for Engagement, was the area that presented lower scores at the T0 assessment. Opportunities for Engagement is described as a parent’s ability to provide choices of objects or activities and their utilization of turn-taking, expectant waiting, blocking, and environmental set-up to create opportunities for social communication (e.g., putting preferred items out of reach or in child-proof containers). Our result on parents’ abilities to provide Opportunities for Engagement is in line with the study by Vibert et al. [50], where raw scores remained below 5 points both at the initial and final assessment. While some behaviors might not be easily elicited during an observation of free play in a clinical setting (e.g., materials were provided without containers and were all spread out on a carpet in front of the child and caregiver), thus penalizing the evaluation of parents’ capacity to set-up the environment, it is possible that this type of strategy might be more challenging for parents to learn. Indeed, they improved throughout the intervention, but needed more time compared to other strategies, and did not completely master this ability by T2. This result is also interesting as it might inform therapists’ work. During the coaching, our trained therapists did not use a didactic or demonstrative approach, but used a “less to more” support, commenting positively on parents’ positive behaviors, and helping them to synchronize themselves better and respond appropriately and timely to their children’s subtle communication signs. Therefore, we did not instruct parents on the use of “blocking techniques” or invite them to put toys away in containers. However, we supported parents in expectant waiting and helped them to propose new activities when the previous activity became too repetitive or the child did not spontaneously choose a new activity. A strategy parents often found challenging to implement, given their children’s specific difficulties, was turn-taking, as some became overly passive, while others tended to overstimulate their children. Globally, however, the parents that underwent our intervention achieved a good fidelity level at the final assessment (M: 81.5%), indicating good management of NDBI strategies. This result is in accordance with Brown et al.’s study [50] that analyzed parents’ strategy use across multiple NDBIs and involved caregiver-mediated approaches; the study found parents improved or maintained high skills in their strategy use (measured with the MONSI-CC) over the course of the intervention.

As described in Brown’s study [52], we expected that participation in a parent-mediated intervention would have a positive impact on children’s social communication development. Indeed, children participating in our study gained significant improvements in all domains of the intervention curriculum (ESDM checklist), both at T1 and at T2. It is noteworthy that improvements in the social and communication domains were confirmed by reduced severity scores in the Social Communication domain of the BOSCC. The BOSCC [51] is a treatment response measure developed to assess changes in core ASD symptoms in young children. In accordance with our expectations, an intervention mediated by parents can have a significant impact on children’s core ASD symptoms. As we observed for parents’ use of NDBI strategies, we found a significant change in children’s Social Communication domain and Restricted and Repetitive Behaviors domain at the BOSCC, after only 3 months of intervention. Importantly, these improvements were maintained at T2, and the difference in BOSCC Core Total scores between the start and end of treatment (M= −5.27) was similar to that identified for treatment responders in previous studies [51,53]. This is a meaningful result as children were not receiving any other intervention, and the intervention itself was of low intensity (1 h per week).

A limitation of this study is that we cannot draw conclusions about the efficacy of the intervention as these preliminary analyses did not include data from a control group. The absence of a comparison group limits our ability to determine whether the observed changes are attributable to the intervention itself or to other factors such as maturation, external influences, or measurement variability. Additionally, the small sample size (n = 20) limits the generalizability of our findings and increases the risk of Type I and Type II errors. Another limitation is the absence of long-term follow-up, which prevents conclusions about the durability of the observed effects over time. Finally, the study focused on a limited set of outcome measures, and the lack of systematic evaluation of broader domains such as adaptive functioning, language development, or parental stress limits our understanding of the intervention’s overall impact. However, for the purpose of investigating the feasibility of this approach, we can conclude that participation was consistent and that parents were able to acquire a good level of fidelity in the use of NDBI strategies. The impact on children’s core ASD symptoms appeared positive, although more rigorous studies are needed to evaluate the efficacy of the model.

A point of merit of this study is that it was applied in a community service, without a stringent use of inclusion criteria, and thus it is representative of ASD children that typically reach public services. Another strength of this study is that the measures reported were double rated by two coders who did not take part in the intervention and were unaware of the time point of each recorded interaction, i.e., they did not know if they were scoring an initial or final assessment.

Our promising results are in line with international and national guidelines on ASD intervention [25,26,27], which highly recommend parents’ involvement. Not only did parents benefit from our intervention, but their children also showed improvements in their core ASD symptoms, demonstrating the importance of parents’ active involvement in their children’s intervention.

Having established the feasibility of implementing a parent-mediated intervention in an Italian community setting, the next step is to rigorously evaluate its efficacy in a larger cohort, using a randomized controlled trial to ensure robust and generalizable results. Furthermore, long-term follow-up will be essential to determine the durability of the observed effects over time and to assess potential implications for healthcare costs.

## Figures and Tables

**Table 1:** Child demographic and baseline characteristics.

Children’s Characterization	M (SD)	
*n*	20	
M/F	15/5	
Age (months)	28.40 (8.26)	
Autism Assessment		
ADOS-2 CSS-Total	6.38	(1.71)
Cognitive Measure		
Griffiths-III General Quotient	73.85	(19.10)
Adaptive Behavior		
Vineland-II Composite Scale	82.44	(15.27)

**Table 2:** Parents’ demographic information.

**Age at Start of Intervention (Years)**	**Mothers**	**Fathers**
M (SD)	36.35 (5.80)	39.65 (6.44)
Range	29–44	28–52
**Educational Level (%)**	**Mothers**	**Fathers**
Doctoral degree	0	10%
University degree	45%	25%
High school diploma	50%	65%
Middle school diploma	5%	0

**Table 3:** Changes over time in MONSI-CC subdomains assessed by repeated measures ANOVA.

MONSI-CC	T0		T1		T2		ANOVA		Post Hoc Comparisons					
									T0–T1		T0–T2		T1–T2	
	M	(SD)	M	(SD)	M	(SD)	F	*p* Value	t	*p* Value	t	*p* Value	t	*p* Value
Environmental Set-up	8.76	(2.53)	12.00	(1.17)	12.66	(2.25)	48.03	<0.001 ***	−5.50	<0.001 ***	−5.58	<0.001 ***	−1.35	0.580
Child-Guided Interactions	17.50	(3.10)	21.03	(2.46)	21.47	(4.68)	24.34	<0.001 ***	−4.05	<0.001 ***	−4.50	<0.001 ***	−0.46	1.000
Active Teaching	23.11	(3.80)	27.66	(3.43)	28.48	(3.13)	25.46	<0.001 ***	−4.90	<0.001 ***	−7.85	<0.001 ***	−1.02	0.966
Opportunities for Engagement	2.71	(0.81)	3.42	(0.98)	3.87	(1.40)	6.41	0.004 **	−2.18	0.108	−3.55	0.003 **	−1.38	0.536
Reinforcing and Scaffolding	11.48	(2.20)	13.08	(1.76)	14.00	(0.96)	12.92	<0.001 ***	−2.68	0.046 *	−5.14	<0.001 ***	−2.32	0.098
Total Score	63.11	(10.28)	77.19	(7.50)	81.48	(6.03)	48.03	<0.001 ***	−7.18	<0.001 ***	−9.37	<0.001 ***	−0.53	0.106

* *p* < 0.05; ** *p* < 0.01; *** *p* < 0.001. Note: *p*-value adjusted for comparing a family of 3.

**Table 4:** Changes over time in the ESDM Curriculum domains assessed by repeated measures ANOVA.

ESDM Checklist	T0		T1		T2		ANOVA		Post Hoc Comparisons					
									T0–T1		T0–T2		T1–T2	
	M	(SD)	M	(SD)	M	(SD)	F	*p* Value	t	*p* Value	t	*p* Value	t	*p* Value
Communication Domain	49.78	(26.99)	81.83	(30.65)	105.67	(37.60)	89.06	<0.001 ***	−7.63	<0.001 ***	−13.30	<0.001 ***	−5.67	<0.001 ***
Socialization Domain	35.79	(15.88)	63.28	(24.92)	71.56	(29.18)	32.75	<0.001 ***	−5.94	<0.001 ***	−7.73	<0.001 ***	−1.78	0.248
Cognition and Play Domain	24.39	(14.92)	41.50	(18.55)	49.61	(19.92)	47.72	<0.001 ***	−6.49	<0.001 ***	−9.57	<0.001 ***	−3.08	0.012 *
Motor Domain	37.11	(18.83)	57.39	(22.42)	68.11	(21.91)	78.12	<0.001 ***	−8.05	<0.001 ***	−12.31	<0.001 ***	−4.26	<0.001 ***
Total Domains Score	147.06	(71.76)	244.00	(87.04)	294.94	(104.03)	88.58	<0.001 ***	−8.59	<0.001 ***	−13.11	<0.001 ***	−4.52	<0.001 ***

* *p* < 0.05; *** *p* < 0.001. Note: *p*-value adjusted for comparing a family of 3.

**Table 5:** Changes over time in BOSCC domains assessed by repeated measures ANOVA.

BOSCC	T0		T1		T2		ANOVA		Post Hoc Comparisons					
									T0–T1		T0–T2		T1–T2	
	M	(SD)	M	(SD)	M	(SD)	F	*p* Value	t	*p* Value	t	*p* Value	t	*p* Value
Social Communication	22.08	(7.90)	18.61	(7.08)	18.34	(8.75)	3.37	0.046 *	2.16	0.065	2.33	0.065	0.16	0.872
Restricted and Repetitive Behaviors	6.24	(2.98)	4.61	(2.77)	4.71	(2.82)	4.59	0.017 *	2.71	0.031 *	2.53	0.032 *	−0.18	0.862
Total Score	28.32	(10.19)	23.21	(9.10)	23.05	(10.93)	4.57	0.029 *	2.58	0.035 *	2.66	0.035 *	0.08	0.944

* *p* < 0.05; Note: *p*-value adjusted for comparing a family of 3.

## Data Availability

The data presented in this study are not publicly available due to privacy restrictions; however, they are available upon request from the corresponding author.

## Abbreviations

The following abbreviations are used in this manuscript:

ADOS-2	Autism Diagnostic Observation Schedule-Second Edition
ASD	Autism Spectrum Disorder
BOSCC	Brief Observation of Social Communication Change
CPMT	Cooperative Parent-Mediated Therapy
CST	Caregiver Skills Training
eTAU	enhanced Treatment-As-Usual
LPI	Low-intensity Psychosocial Intervention
MONSI-CC	Measure of NDBI Strategy Implementation: Caregiver Change
NDBI	Naturalistic Developmental Behavioral Intervention
Parent-ESDM	Parent-mediated Early Start Denver Model
SC	Social Communication
RRB	Restricted and Repetitive Behaviors

## References

[1] American Psychiatric Association (2022). Diagnostic and Statistical Manual of Mental Disorders.

[2] Marsack-Topolewski C.N., Wang F., Samuel P.S. (2025). Characteristics of adult children with autism and caregiver burden. Fam. Relat..

[3] Leigh J.P., Du J. (2015). Brief report: Forecasting the economic burden of autism in 2015 and 2025 in the United States. J. Autism Dev. Disord..

[4] Zhao Y., Lu F., Wang X., Luo Y., Zhang R., He P., Zheng X. (2024). The economic burden of autism spectrum disorder with and without intellectual disability in China: A nationwide cost-of-illness study. Asian J. Psychiatr..

[5] Guthrie W., Wetherby A.M., Woods J., Schatschneider C., Holland R.D., Morgan L., Lord C.E. (2023). The earlier the better: An RCT of treatment timing effects for toddlers on the autism spectrum. Autism.

[6] Lombardo M.V., Busuoli E.M., Schreibman L., Stahmer A.C., Pramparo T., Landi I., Mandelli V., Bertelsen N., Barnes C.C., Gazestani V. (2021). Pre-treatment clinical and gene expression patterns predict developmental change in early intervention in autism. Mol. Psychiatry.

[7] Cleary D.B., Maybery M.T., Green C., Whitehouse A.J. (2023). The first six months of life: A systematic review of early markers associated with later autism. Neurosci. Biobehav. Rev..

[8] Dawson G., Rieder A.D., Johnson M.H. (2023). Prediction of autism in infants: Progress and challenges. Lancet Neurol..

[9] Pierce K., Gazestani V.H., Bacon E., Barnes C.C., Cha D., Nalabolu S., Lopez L., Moore A., Pence-Stophaeros S., Courchesne E. (2019). Evaluation of the diagnostic stability of the early autism spectrum disorder phenotype in the general population starting at 12 months. JAMA Pediatr..

[10] Shaw K.A., Williams S., Patrick M.E., Valencia-Prado M., Durkin M.S., Howerton E.M., Ladd-Acosta C.M., Pas E.T., Bakian A.V., Bartholomew P. (2025). Prevalence and Early Identification of Autism Spectrum Disorder Among Children Aged 4 and 8 Years—Autism and Developmental Disabilities Monitoring Network, 16 Sites, United States. MMWR Surveill. Summ..

[11] Payakachat N., Tilford J.M., Kuhlthau K.A. (2018). Parent-Reported Use of Interventions by Toddlers and Preschoolers with Autism Spectrum Disorder. Psychiatr. Serv..

[12] Safonicheva O., Ovchinnikova M. (2020). New approaches to the development of personalized rehabilitation programs for the children with disabilities, including autism spectrum CHAT disorders. Autism 360°.

[13] Chaidi I., Drigas A. (2020). Parents’ involvement in the education of their children with Autism: Related research and its results. Int. J. Emerg. Technol. Learn. (IJET).

[14] Salomone E., Beranová T.P., Bonnet-Brilhault F., Briciet Lauritsen M., Budisteanu M., Buitelaar J., Canal-Bedia R., Felhosi G., Fletcher-Watson S., Freitag C. (2016). Use of early intervention for young children with autism spectrum disorder across Europe. Autism.

[15] Oono I.P., Honey E.J., McConachie H. (2013). Parent-mediated early intervention for young children with autism spectrum disorders (ASD). Evid.-Based Child Health.

[16] Rogers S.J., Estes A., Lord C., Vismara L., Winter J., Fitzpatrick A., Guo M., Dawson G. (2012). Effects of a brief Early Start Denver Model (ESDM)–based parent intervention on toddlers at risk for autism spectrum disorders: A randomized controlled trial. J. Am. Acad. Child Adolesc. Psychiatry.

[17] Ingersoll B., Frost K.M., Straiton D., Ramos A.P., Casagrande K. (2024). Telehealth coaching in Project ImPACT indirectly affects children’s expressive language ability through parent intervention strategy use and child intentional communication: An RCT. Autism Res..

[18] Cheng W.M., Smith T.B., Butler M., Taylor T.M., Clayton D. (2023). Effects of Parent-Implemented Interventions on Outcomes of Children with Autism: A Meta-Analysis. J. Autism. Dev. Disord..

[19] Sigman M., Ungerer J.A. (1984). Attachment behaviors in autistic children. J. Autism Dev. Disord..

[20] Green J., Charman T., Pickles A., Wan M.W., Elsabbagh M., Slonims V., Taylor C., McNally J., Booth R., Gliga T. (2015). Parent-mediated intervention versus no intervention for infants at high risk of autism: A parallel, single-blind, randomised trial. Lancet Psychiatry.

[21] Green J., Pickles A., Pasco G., Bedford R., Wan M.W., Elsabbagh M., Slonims V., Gliga T., Jones E., Cheung C. (2017). Randomised trial of a parent-mediated intervention for infants at high risk for autism: Longitudinal outcomes to age 3 years. J. Child Psychol. Psychiatry.

[22] Bradshaw J., Wolfe K., Hock R., Scopano L. (2022). Advances in supporting parents in interventions for autism spectrum disorder. Pediatr. Clin. N. Am..

[23] Estes A., Vismara L., Mercado C., Fitzpatrick A., Elder L., Greenson J., Lord C., Munson J., Winter J., Young G. (2014). The impact of parent-delivered intervention on parents of very young children with autism. J. Autism Dev. Disord..

[24] Gao D., Yu T., Li C.-L., Jia F.-Y., Li H.-H. (2020). Effect of parental training based on Early Start Denver Model combined with intensive training on children with autism spectrum disorder and its impact on parenting stress. Zhongguo Dang Dai Er Ke Za Zhi Chin. J. Contemp. Pediatr..

[25] World Health Organization Meeting Report: Autism Spectrum Disorders and Other Developmental Disorders: From Raising Awareness to Building Capacity. https://iris.who.int/handle/10665/103312.

[26] Istituto Superiore di Sanità Raccomandazioni della Linea Guida Sulla Diagnosi e sul Trattamento del Disturbo dello Spettro Autistico in Bambini e Adolescenti. https://www.iss.it/en/-/raccomandazioni-lg-diagnosi-trattamento-di-bambini-adolescenti-con-asd.

[27] National Institute for Health and Care Excellence Autism Spectrum Disorder in Under 19s: Support and Management. https://www.nice.org.uk/guidance/cg170.

[28] Borgi M., Ambrosio V., Cordella D., Chiarotti F., Venerosi A. (2019). Nationwide survey of healthcare services for autism spectrum disorders (ASD) in Italy. Adv. Neurodev. Disord..

[29] Colombi C., Narzisi A., Ruta L., Cigala V., Gagliano A., Pioggia G., Siracusano R., Rogers S.J., Muratori F., Team P.P. (2018). Implementation of the early start Denver model in an Italian community. Autism.

[30] Devescovi R., Colonna V., Dissegna A., Bresciani G., Carrozzi M., Colombi C. (2021). Feasibility and outcomes of the early start Denver model delivered within the public health system of the Friuli Venezia Giulia Italian region. Brain Sci..

[31] Valeri G., Casula L., Menghini D., Amendola F.A., Napoli E., Pasqualetti P., Vicari S. (2020). Cooperative parent-mediated therapy for Italian preschool children with autism spectrum disorder: A randomized controlled trial. Eur. Child Adolesc. Psychiatry.

[32] Carta A., Casula L., Manca S., Puci M.V., Puseddu G., Fucà E., Sotgiu G., Vicari S., Sotgiu S., Valeri G. (2025). Cooperative Parent-Mediated Therapy for Italian Children with Autism Spectrum Disorder: A Clinical Experimental Study in a Community Healthcare Service. Front. Child Adolesc. Psychiatry.

[33] Salomone E., Settanni M., McConachie H., Suma K., Ferrara F., Foletti G., Salandin A., Servili C., Adamson L.B., Team W.C. (2022). Pilot Randomized Controlled Trial of the WHO Caregiver Skills Training in Public Health Services in Italy. J. Autism Dev. Disord..

[34] Pellecchia M., Mandell D.S., Beidas R.S., Dunst C.J., Tomczuk L., Newman J., Zeigler L., Stahmer A.C. (2023). Parent coaching in early intervention for autism spectrum disorder: A brief report. J. Early Interv..

[35] Sandbank M., Bottema-Beutel K., Woynaroski T. (2021). Intervention Recommendations for Children with Autism in Light of a Changing Evidence Base. JAMA Pediatr..

[36] Rogers S.J., Dawson G. (2010). Early Start Denver Model for Young Children with Autism: Promoting Language, Learning, and Engagement.

[37] Wang Z., Loh S.C., Tian J., Chen Q.J. (2022). A meta-analysis of the effect of the Early Start Denver Model in children with autism spectrum disorder. Int. J. Dev. Disabil..

[38] Devescovi R., Monasta L., Mancini A., Bin M., Vellante V., Carrozzi M., Colombi C. (2016). Early diagnosis and Early Start Denver Model intervention in autism spectrum disorders delivered in an Italian Public Health System service. Neuropsychiatr. Dis. Treat..

[39] Contaldo A., Colombi C., Pierotti C., Masoni P., Muratori F. (2020). Outcomes and moderators of Early Start Denver Model intervention in young children with autism spectrum disorder delivered in a mixed individual and group setting. Autism.

[40] Cucinotta F., Ricciardello A., Turriziani L., Calabrese G., Briguglio M., Boncoddo M., Bellomo F., Tomaiuolo P., Martines S., Bruschetta M. (2020). FARP-1 deletion is associated with lack of response to autism treatment by early start denver model in a multiplex family. Mol. Genet. Genom. Med..

[41] Devescovi R., Bresciani G., Colonna V., Carrozzi M., Dissegna A., Celea M.A., Cescon D., Frisari S., Guerrieri M., Placer F. (2023). Short-term outcomes of an ESDM intervention in Italian children with Autism Spectrum Disorder following the COVID-19 lockdown. Children.

[42] Vismara L.A., McCormick C.E.B., Wagner A.L., Monlux K., Nadhan A., Young G.S. (2016). Telehealth Parent Training in the Early Start Denver Model: Results from a Randomized Controlled Study. Focus Autism Other Dev. Disabil..

[43] Jhuo R.-A., Chu S.-Y. (2022). A review of parent-implemented early start denver model for children with autism spectrum disorder. Children.

[44] Rogers S.J., Stahmer A., Talbott M., Young G., Fuller E., Pellecchia M., Barber A., Griffith E. (2022). Feasibility of delivering parent-implemented NDBI interventions in low-resource regions: A pilot randomized controlled study. J. Neurodev. Disord..

[45] Strisciuglio P., Griffiths R. (2016). Griffiths III—Scale Griffiths dello Sviluppo del Bambino.

[46] Lord C. (2012). Autism Diagnostic Observation Schedule (ADOS-2) Manual (Part I): Modules 1–4.

[47] Lord C. (2012). Autism Diagnostic Observation Schedule (ADOS-2) Manual (Part II): Toddler Module.

[48] Colombi C., Tancredi R., Persico A., Faggioli R. (2013). ADOS-2—Autism Diagnostic Observation Schedule.

[49] Sparrow S.S., Cicchetti D.V., Balla D.A. (2005). Vineland Adaptive Behavior Scales.

[50] Vibert B.A., Dufek S., Klein C.B., Choi Y.B., Winter J., Lord C., Kim S.H. (2020). Quantifying Caregiver Change Across Early Autism Interventions Using the Measure of NDBI Strategy Implementation: Caregiver Change (MONSI-CC). J. Autism Dev. Disord..

[51] Grzadzinski R., Carr T., Colombi C., McGuire K., Dufek S., Pickles A., Lord C. (2016). Measuring changes in social communication behaviors: Preliminary development of the Brief Observation of Social Communication Change (BOSCC). J. Autism Dev. Disord..

[52] Brown P., Soulé Z., Whitman G. (2025). Determining VO. Nutr. Clin. Pract..

[53] Carruthers S., Charman T., El Hawi N., Kim Y.A., Randle R., Lord C., Pickles A., Consortium P. (2021). Utility of the autism diagnostic observation schedule and the brief observation of social and communication change for measuring outcomes for a parent-mediated early autism intervention. Autism Res..

